# Effectiveness of Low-Dose Atropine Combined With Bright Light Therapy for Controlling Myopic Eye Growth in Schoolchildren: Study Protocol for a Randomized Controlled Trial

**DOI:** 10.2196/90893

**Published:** 2026-04-13

**Authors:** Dennis Yan-Yin Tse, Martin Ming-Leung Ma, Ying Hon, Lai Ming Ho, Wai Ching Lam, Kendrick Co Shih, Xiujuan Zhang, Desmond Man Kit Lam, Andy Chi Wai Kong, Thomas Chuen Lam, Carly Siu Yin Lam, Tina Jinxiao Lian, Ian Morgan, Chi Ho To, Rachel Ka Man Chun, Christopher Kai Shun Leung

**Affiliations:** 1 Research Centre for SHARP Vision, The Hong Kong Polytechnic University, Hong Kong, China (Hong Kong); 2 InnoHK, Centre for Eye and Vision Research, 17W Hong Kong Science Park, Hong Kong, China (Hong Kong); 3 Centre for Myopia Research, School of Optometry, The Hong Kong Polytechnic University, 11 Yuk Choi Rd, Hung Hom, Kowloon, China (Hong Kong), 852 27666096; 4 Vancouver Coastal Health Research Institute, Vancouver, Canada; 5 School of Public Health, The University of Hong Kong, Hong Kong, China (Hong Kong); 6 Department of Ophthalmology, The University of Hong Kong, Hong Kong, China (Hong Kong); 7 Research School of Biology, Australian National University, Canberra, Australia

**Keywords:** myopia control, bright light, atropine, refractive errors, myopia, clinical trial, vision

## Abstract

**Background:**

Myopia is increasingly prevalent worldwide, with projections indicating that nearly 50% of the global population may have myopia by 2050. This surge poses significant concerns due to its impact on vision and quality of life and its link to a range of blinding diseases, including myopic macular degeneration, glaucoma, and retinal detachment. Current pharmacologic and optical interventions offer limited effectiveness in slowing myopia progression, highlighting the urgent need for more effective treatments.

**Objective:**

This study aims to examine the combined effect of bright light therapy and low-dose atropine on myopic progression.

**Methods:**

This is a single-site, 2-arm, single-masked (examiner-masked) randomized controlled trial to compare the effectiveness of low-dose atropine alone and its combination with bright light therapy in retarding myopia progression. The study protocol has been approved by the institutional review boards of Hong Kong Polytechnic University (HSEARS 20180829002-05) and the University of Hong Kong and Hospital Authority Hong Kong West Cluster (UW 20-362). Schoolchildren with myopia aged 7 to 12 years who have not undergone any previous myopic control intervention will be recruited and randomly allocated into 2 groups (n=67 per group) after baseline measurements. Both groups will receive 0.01% atropine twice daily for 24 months. The combination treatment group will also receive a high-intensity lamp for bright light therapy. The primary and secondary outcome measures will be the changes in cycloplegic autorefraction in spherical equivalent refraction and axial length, respectively, measured every 6 months over 2 years from baseline.

**Results:**

The project was funded in January 2019. The recruitment process started on March 21, 2023, and was completed on February 2, 2024. Data collection is expected to be completed in April 2026.

**Conclusions:**

This study will provide new information on whether the combination of bright light therapy and low-dose atropine is more effective than atropine alone in slowing down myopia progression. It will also assess the effectiveness of low-dose atropine used twice daily. Combining bright light therapy and atropine could become a new treatment option if shown to be effective. New data on the effectiveness of using atropine twice daily might also expand available treatment options.

## Introduction

Myopia has become increasingly prevalent worldwide, particularly among children in the Asian region. It has been predicted that almost 50% of the world’s population will have myopia by 2050 [1]. This rapid rise in myopia is alarming not only because of its impact on vision and quality of life [2] but also because of its association with a higher risk of developing a range of blinding diseases later in life, including myopic macular degeneration, glaucoma, and retinal detachment [3].

Previous studies have identified pharmacologic and optical methods to retard myopia progression that only have a low to moderate effectiveness [4]. Thus, there is still an urgent need to explore innovative treatment approaches that are more capable than current methods. One promising approach is bright light therapy. Outdoor illumination in direct sunlight can reach up to 100,000 lux, whereas indoor illumination typically ranges from 100 to 500 lux [5]. One hypothesis explaining the rising prevalence of myopia among schoolchildren is that urban populations have embraced a lifestyle that limits exposure to bright outdoor light [6]. Research on animals (chicks, mice, and rhesus monkeys) has demonstrated that exposure to bright light ranging from 15,000 to 40,000 lux can entirely inhibit the development of myopia [7-10]. Observational clinical studies have indicated that an increase in time spent outdoors is associated with a more hyperopic refractive error and a lower incidence of juvenile-onset myopia [11,12]. In addition, a 1-year-long randomized clinical trial by Wu et al [13] has shown that an increase in outdoor activity can lead to less myopic shift and axial elongation in both children with and without myopia. The protective effect against myopia does not appear to be due to physical exercise, as indoor exercise does not inhibit myopia [14].

While it is clear that spending more time outdoors is beneficial for preventing and controlling myopia, the next clinically relevant question is whether artificially applied bright light indoors could achieve the same effect. Therefore, our team has designed a randomized controlled study to investigate the effect of bright light therapy (approximately 10,000 lux) on myopia [15]. This innovative approach will also be examined to explore the effect of its combination with low-dose atropine.

As no current treatment has been found to be able to completely halt myopia progression, another option is the potential use of a combination treatment. It is hypothesized that bright light therapy could provide additive efficacy to slow myopia progression when used in combination with low-dose atropine. Low-dose atropine has demonstrated a dose-dependent effect on slowing myopia with minimal side effects [16]. This combination is promising as there are preliminary data suggesting that these 2 therapies work on different mechanisms. While the exact mechanism for atropine’s action is still unknown, it is thought to affect the development of myopia mainly by acting as a reversible competitive antagonist of acetylcholine muscarinic receptors that are found in many parts of the eye [17]. Regarding bright light therapy, its inhibitory effect on myopia is suggested to be mediated by the retinal dopaminergic pathways [18].

On the basis of these rationales, this clinical trial was designed to examine the combined effect of bright light therapy and low-dose atropine on myopic progression. The primary aim of this trial is to investigate whether the combination treatment with bright light therapy and low-dose atropine would be more effective than atropine alone. This trial will also assess the effectiveness of low-dose atropine in slowing myopia progression. Factors associated with the effectiveness of the treatments will also be identified. In addition, compliance and participant acceptability (particularly in terms of visual performance) of the combination therapy will be evaluated.

## Methods

### Study Design and Setting

**Figure 1.**
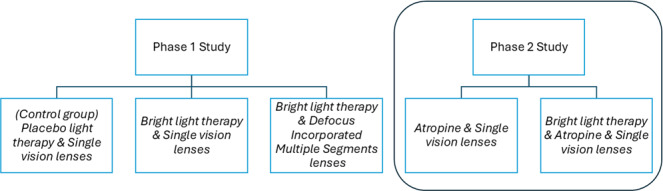
Intervention groups in the phase 1 and phase 2 studies. The protocol for the phase 1 study has been published elsewhere [15]. This paper only describes the design of the phase 2 study.

This protocol describes a single-site, 2-arm, single-masked (examiner-masked) randomized controlled trial comparing the effectiveness of low-dose atropine alone and in combination with bright light therapy in retarding myopia progression. This manuscript is based on protocol version 1.6 (September 2022). This study will last for 2 years and is an auxiliary study to a previous study (phase 1 study) [15] conducted by the same team sharing the same study design except for the choice of treatment. The phase 1 study [15] will consist of 1 control group and 2 treatment groups, whereas this phase 2 study will consist of 2 treatment groups only. This paper will only describe the design of the phase 2 study, and details of the intervention for each group are shown in Figure 1 [15]. The investigation will be separated into 2 phases due to logistical issues. Participants from the phase 1 study will not continue into the phase 2 study. This study consists of 2 arms: atropine and atropine combined with bright light therapy. Participants will be randomized to the 2 treatment groups at a ratio of 1:1 using an age-stratified simple randomization. Data from the 2 phase 2 treatment groups will be compared with each other, and comparisons with phase 1 data will be conducted for exploratory analysis. Identical inclusion criteria will be used for both studies to minimize potential selection bias. The application of consistent methodologies and statistical approaches will enable meaningful comparisons across interventions.

All data collection will take place at the Optometry Research Clinic of Hong Kong Polytechnic University or the HKU Eye Centre, Department of Ophthalmology, University of Hong Kong. This trial protocol is reported in accordance with the SPIRIT (Standard Protocol Items: Recommendations for Interventional Trials) guidelines [19].

### Ethical Considerations

This study protocol meets the tenets of the Declaration of Helsinki and has been approved by the institutional review boards of Hong Kong Polytechnic University (HSEARS 20180829002-05) and the University of Hong Kong and Hospital Authority Hong Kong West Cluster (UW 20-362). Written consent from the participants and their parents and assent from the participants will be obtained by examiners before any study procedures are performed. The study was prospectively registered at ClinicalTrials.gov (NCT04923841) on June 11, 2021. All procedures will adhere to the good clinical practice guidelines. Study personnel, including administrative staff and investigators, will complete the good clinical practice certification course. Patients and members of the public were not involved in the design, conduct, reporting, or dissemination planning of this research. After publication, parents or guardians and participants will be informed of the study results via a newsletter. This paper does not contain any individual identifiable data.

### Sample Size Calculation

The sample size is estimated based on the 2-year change in refractive error in the 0.01% atropine treatment group (−0.64, –0.44 to +0.44 D) from a previous myopia control study [20]. Assuming that, over 2 years, combination treatment is more effective than 0.01% atropine alone by 0.25 D, which is considered a clinically significant change, 56 participants per group will be required. This sample size will enable the demonstration of a statistically significant difference between the 2 groups at a 5% significance level with 85% power. The sample size for each study arm will be adjusted to 67 per arm assuming a 20% loss to follow-up. As a result, a total sample size of 134 participants will be recruited for this study.

### Inclusion Criteria

Children meeting the following criteria will be included:

- Children who are Hong Kong Chinese
- Myopia (spherical equivalent) of −0.75 D to −5.00 D in both eyes
- Age at enrollment of 7 to 12 years
- Astigmatism and anisometropia of 1.50 D or less
- Spectacle-corrected monocular visual acuity of 0.04 logMAR or better in both eyes
- Normal binocular function
- Parents’ understanding and acceptance of the random allocation of grouping and masking
- Ability to wear the prescribed spectacles, apply eye drops, and undergo light therapy daily

### Exclusion Criteria

Children meeting the following criteria will be excluded:

- Eye disease or binocular vision problems (eg, strabismus, amblyopia, oculomotor nerve palsies, or corneal disease)
- Previous intraocular or corneal surgery
- Ocular or systemic disease that may affect vision function or development (eg, endocrine, cardiac, and respiratory diseases; diabetes; or Down syndrome)
- Allergy to atropine
- Previous or current use of gas-permeable, soft bifocal, or orthokeratology contact lenses or bifocal, progressive addition lenses; specific myopic control spectacle wear; or use of atropine or pirenzepine (longer than 1 month)
- Previous or current participation in myopia control studies

### Randomization and Masking

Participants will be randomized to the 2 treatment groups at a ratio of 1:1 using stratified simple randomization. The randomization list will be generated using a random number generator in a Microsoft Excel spreadsheet. Participants will be stratified into 2 age groups (7-9 years and 10-12 years). This is a single-masked study in which examiners are masked to the treatment allocation. Allocation concealment will be ensured using sequentially numbered, opaque, sealed envelopes. The envelopes will be opened by the designated unmasked investigators in numerical order only after the participants are enrolled. To maintain masking, access to the randomization list will be limited to the trial steering committee; study management committee; statistician; and unmasked investigators designated to handle randomization, allocation, and delivery of group assignments. These individuals will not be involved in participant enrollment or data collection.

### Procedures

Mass emails to the staff of Hong Kong Polytechnic University, advertising posts on social media (Facebook and Instagram), and referrals from parents or guardians who are already participating in the study will be used to recruit participants. Parents or guardians and their children will be invited to the Optometry Research Clinic for a screening visit to confirm their eligibility. Informed consent and assent will be obtained upon confirmation of fulfillment of the inclusion criteria.

Successfully enrolled participants will be randomly allocated to 1 of the 2 arms after baseline measurement. Delivery of spectacles and a light therapy lamp will be arranged 2 weeks after the baseline visit. Training will be provided to participants and parents or guardians on how to set up and use the light therapy lamp at home. The participants in both treatment groups will be asked to return 1 month after the intervention for aftercare appointments to ensure that there are no allergic reactions to atropine or adverse events associated with the light therapy lamp. Participants will also be asked to return for regular follow-up visits with the study ophthalmologist for dispensing of 0.01% atropine.

Data collection will be conducted every 6 months from the baseline visit up to 24 months. Baseline measurements will include demographic data; medical and medication history; visual acuity; tests for strabismus, autorefraction, and keratometry; pupil size; amplitude of accommodation; accommodative lag; subjective and objective refraction with and without cycloplegia; intraocular pressure; choroidal thickness; external and internal ocular health; peripheral refraction; and axial length via the IOLMaster 500 (ZEISS) and other ocular biometry (eg, corneal curvature, corneal thickness, anterior chamber depth, and lens thickness) via the Lenstar LS 900 (Haag-Streit AG). In addition, questionnaires on visual performance, daily activities, and compliance will be administered at the 6-, 12-, 18-, and 24-month visits. A SPIRIT schedule of the study is shown in Table 1.

**Table 1.** Schedule of enrollment, interventions, and assessments.

Timepoint	Baseline	1 month	6 months	12 months	18 months	24 months
Enrollment						
Allocation	✓					
Interventions						
Single treatment: atropine & single vision lenses		✓	✓	✓	✓	✓
Combination treatment: bright light therapy & atropine & single vision lenses		✓	✓	✓	✓	✓
Assessments						
Primary outcome: cycloplegic objective refraction	✓		✓	✓	✓	✓
Secondary outcome: axial length	✓		✓	✓	✓	✓
Other outcomes: intraocular pressure, accommodative functions, pupil size, and peripheral refraction	✓		✓	✓	✓	✓
Safety outcomes: treatment compliance, visual performance, and adverse events		✓	✓	✓	✓	✓

### Interventions

Eligible participants will be randomized to 1 of 2 groups: (1) bright light therapy and atropine group (high-intensity lamp for bright light therapy, 0.01% atropine eye drops twice a day [1 drop in the morning and 1 drop at night before bedtime], and single vision spectacle lenses) and (2) atropine group (0.01% atropine eye drops twice a day [1 drop in the morning and 1 drop at night before bedtime] and single vision spectacle lenses). Participants will stay in these groups for 24 months.

#### Bright Light Therapy

**Figure 2.**
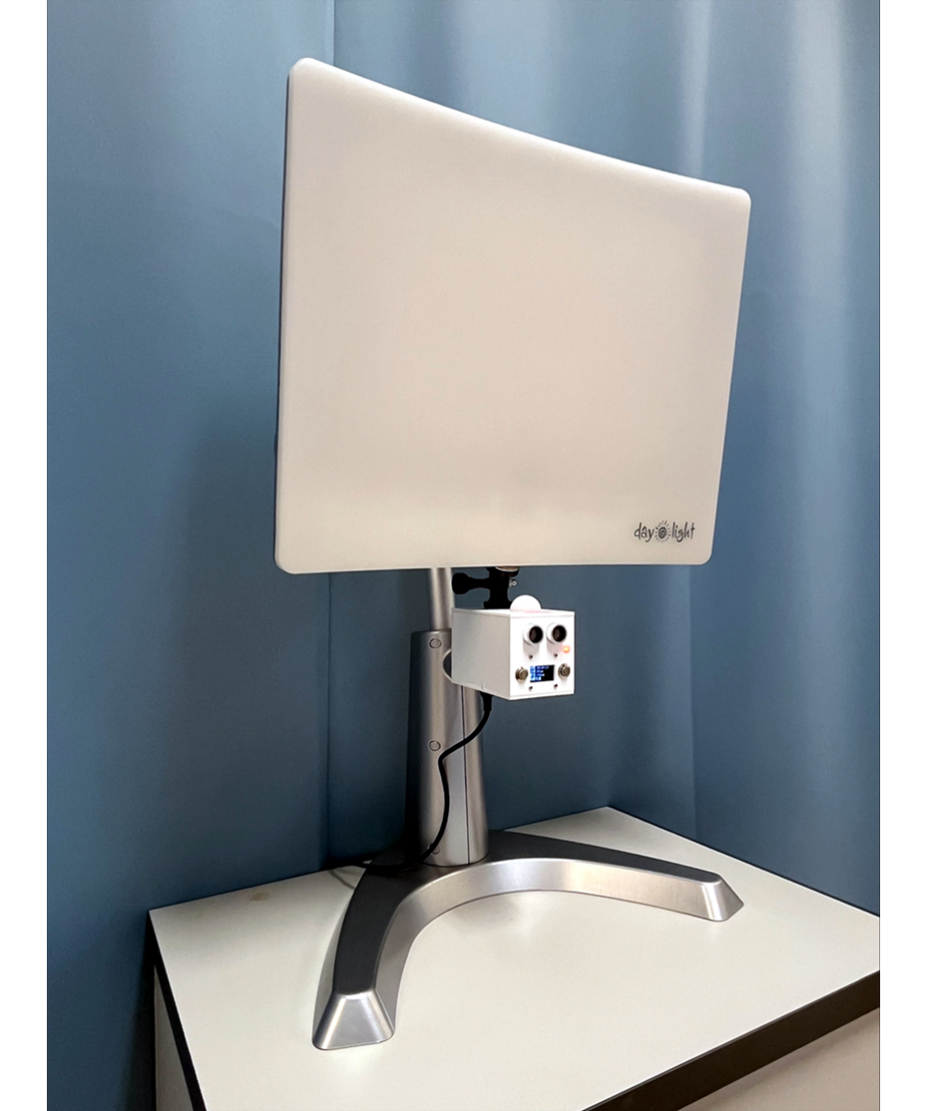
Light therapy lamp with monitoring sensor attached underneath.

**Figure 3.**
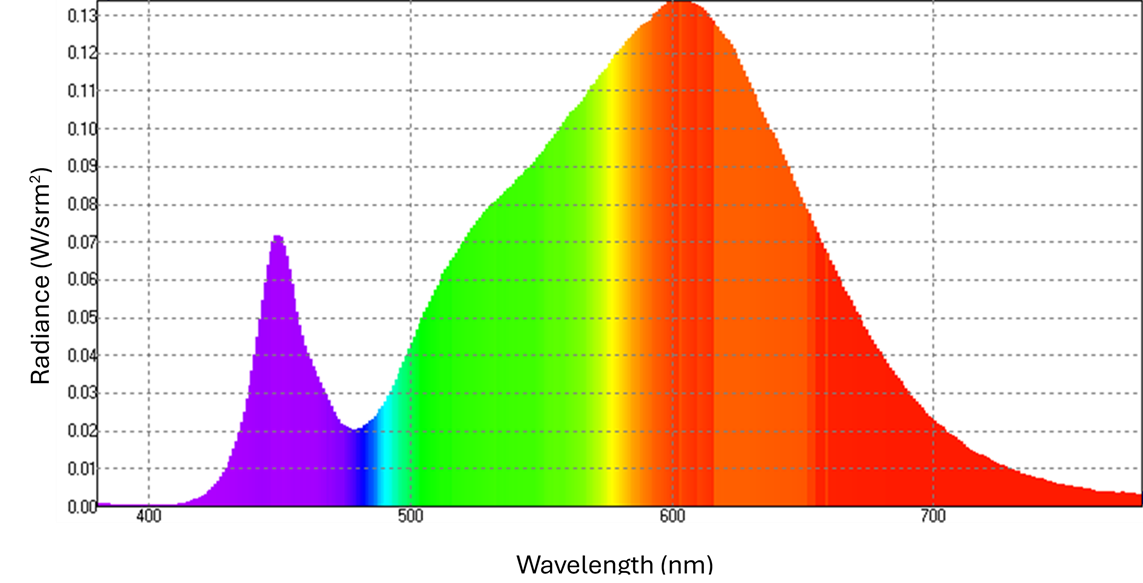
Spectral radiance of the light therapy lamp measured using a spectroradiometer (model S-R3; Topcon Technohouse Co).

During bright light therapy, participants are exposed to an LED lamp on a daily basis as they perform near visual tasks (eg, homework, reading, or using a computer). Each participant will be provided with a lamp (model DL93011, Carex Health Brands; Figure 2) commonly used in previous psychiatric studies [21]. It emits 4000-K glare-free white light rated at 10,000 lux at 30 cm from the screen to the cornea. The spectral radiance of the lamp can be found in Figure 3. The polycarbonate diffuser of the unit blocks UV light with a cutoff of approximately 400 nm. Participants will use the lamp at home and be given standardized verbal instructions, including viewing distance, viewing angle, and duration of use. The lamp head should be angled at approximately 15 degrees from vertical, with the center of the lamp head located 30 to 55 cm from the participant’s eye. Participants will be asked to use the unit for at least 45 minutes per day (not more than 120 minutes per day) and 7 days per week. This dosage is chosen because clinical trials using similar protocols (30-60 minutes per day) have reported effective remission of depressive symptoms and well-tolerated treatment [22]. Participants may choose when to use the lamp during the day and to skip the treatment on days when they have already had more than 2 hours of outdoor activities under sunlight. To avoid causing insomnia, participants will be advised not to use the lamp within 3 hours prior to bedtime. They will also be trained on how to use the unit properly. There will be a network-enabled device attached to the lamp to monitor and record the duration of treatment, working distance, and ambient light illuminance. To promote compliance, researchers will make phone calls to participants (or their guardians) regarding lamp use during the first month of the trial and to provide guidance when needed. All lamps will be returned to investigators at the end of the study.

#### Atropine

Sterile, unpreserved, unidose 0.01% atropine eye drops will be provided to parents or guardians for administering to the participants as 1 drop in the morning and 1 drop at night before bedtime (twice a day) for 24 months. Each vial will be disposed of daily to prevent microbial contamination. An ophthalmologist will perform an ocular health assessment at the atropine dispensing visit, 1 month later, and every 6 months thereafter.

#### Single Vision Spectacle Lenses

The single vision spectacles, prescribed according to the cycloplegic subjective refraction, will be provided to participants in both treatment groups. The lenses are made of plastic, with a refractive index of 1.6. Participants will be instructed to wear the spectacles full time. At each follow-up visit, an update in prescription will be made if either of these criteria is fulfilled: (1) an increase in myopia of 0.50 D (spherical overrefraction) or more or (2) habitual visual acuity equal to or worse than 0.20 logMAR.

### Outcomes and Measurements

The primary and secondary outcome measures will be changes in cycloplegic autorefraction in spherical equivalent refraction and axial length, respectively, measured every 6 months over 2 years from baseline. Other outcomes include peripheral refraction, pupil size, choroidal thickness, amplitude of accommodation, accommodative lag, corneal curvature, noncontact intraocular pressure, visual performance questionnaire, and treatment compliance (assessed via the frequency and/or duration of the use of atropine and bright light therapy).

### Compliance

Compliance with spectacle wear will be monitored through questionnaires at 6-month intervals. Compliance with light therapy will be monitored through 2 means. First, participants or their parents or guardians will be instructed to record the treatment duration daily using a mobile app. Second, a cloud-enabled monitoring sensor attached to the bottom of each light therapy lamp (Figure 2) will be used to collect data on background lighting, working distance, and use time for each participant. Compliance with atropine eye drops will be monitored through a self-reporting log through the aforementioned mobile app. Additionally, custom-built medicine boxes with cloud-enabled sensors will be randomly distributed to 20 participants in the atropine group. The sensors will collect data on the time and frequency of the actions of opening and closing the boxes, providing another measure of compliance.

Compliance data will be analyzed monthly, and participants with poor compliance will be reminded to follow the treatment modality via WhatsApp messages.

### Safety Considerations

The side effects of bright light therapy have been extensively researched in both psychiatric patients and healthy adults [22,23]. No ocular abnormalities have been found with either short- or long-term bright light therapy [24]. Some commonly reported side effects include headaches, eye strain, blurred vision, and nausea, but these were mild and temporary [23,25]. Bright light therapy is generally considered a safe and well-tolerated treatment [22].

A meta-analysis of the safety of low-dose atropine revealed that no major adverse events were detected in all 4 studies included. Side effects of atropine include photophobia, blurred near vision, lower accommodation amplitude, and a larger pupil size [26]. Overall, low-dose atropine is considered a safe treatment for myopia control [26,27].

The visual functions, ocular health, and visual and nonvisual symptoms of both treatment groups will be strictly monitored at every follow-up visit. Adverse events, causally related or not to the study intervention, will be recorded and classified by severity. These include but are not limited to reduced visual acuity, visual disturbances, eye strain, eye infection or allergic reaction, photophobia, headache, or nausea. Participants will receive appropriate medical care for any clinically significant adverse events. Any serious adverse events will be reported to the Institutional Review Board of Hong Kong Polytechnic University within 48 hours. All adverse events will be followed up on until adequate resolution. An investigator may withdraw a participant from the study if any clinical adverse event or situation occurs such that continued participation in the study would not be in their best interest.

### Data Management Plan

The source documents consist of signed consent and assent forms, printouts of ophthalmic investigations (eg, autorefractor and tonometer), and electronic case report forms (eCRFs). All paper documents will be stored in lockable cabinets. All study data will be recorded on standardized eCRFs and stored in an online REDCap (Research Electronic Data Capture; Vanderbilt University) [28] database, in which the server is built and protected under a secure campus network. Access is restricted to authorized study investigators, each with a unique account, ID number, and password. Only specific forms can be viewed and modified based on the investigators’ assigned roles. Anonymized participant identifiers will be used for any data entry and documents uploaded to REDCap. An unmasked investigator must review and approve each eCRF after all data have been entered. An independent monitor will check the presence and correct date of all signed consent forms. The monitor will sample approximately 10% of all randomized participants to check correct data collection and data entry for the key points of this study.

### Oversight and Monitoring

The trial steering committee comprises DYYT (chair), CHT (co–principal investigator), LMH (co–principal investigator), and Professor Chea-su Kee (departmental research committee chair of the School of Optometry, Hong Kong Polytechnic University). The study management committee comprises DYYT (principal coordinator), CKSL (co–principal investigator), CSYL (co–principal investigator), and Professor Victor Chi-Pang Woo (external member from Hong Kong Polytechnic University). The day-to-day coordination of the trial will be managed by the study team at the School of Optometry, Hong Kong Polytechnic University, and the Department of Ophthalmology, University of Hong Kong.

### Statistical Analyses

The latest version of the SPSS software (IBM Corp) will be used for statistical analyses. This study will use the same statistical approach as that used in the previous phase 1 study [15]. Data from the right eyes will be used for analyses. The normality of the data will be examined using the Kolmogorov-Smirnov test. Demographic and optometric data will be presented as means and SDs or as numbers and percentages as appropriate. The chi-square test (or, if appropriate, the Fisher exact test) and one-way ANOVA will be performed to compare baseline between-group differences in demographics (age and sex). Changes in primary, secondary, and other outcomes at different time points will be analyzed using a mixed-effects model for repeated measures, which includes fixed effects for the treatment group, time, the interaction between group and time, and covariates including age and baseline myopia. If statistically significant differences in baseline demographic characteristics between arms are found, these will also be adjusted for.

The progression of myopia over 2 years will be calculated as the difference in cycloplegic autorefraction in spherical equivalent refraction and axial length between the baseline and the 2-year visit. The relative treatment effect in each arm will be determined by dividing the difference in myopia progression between the treatment group and the control group in the phase 1 study [15] by myopia progression in the control group and then multiplying by 100%. Additionally, the absolute treatment effect [29] will be presented to facilitate comparison with other studies. Multiple regression analysis will be used to identify factors associated with myopia control effectiveness.

All analyses will follow the intention-to-treat approach. Missing values of outcome variables and covariates will be replaced using a multiple imputation procedure with 10 sets of imputations assuming missingness at random. Sensitivity analyses for the primary outcomes include (1) complete-case analysis and (2) per-protocol analysis. All analyses will be 2-tailed, with a significance level of 5%.

## Results

The project was funded in January 2019. The recruitment process started on March 21, 2023, and was completed on February 2, 2024. Data collection is expected to be completed in April 2026. The results are expected to be published in winter 2026.

## Discussion

This 2-arm randomized controlled trial will be the first to investigate the combined effect of bright light therapy and low-dose atropine on myopia progression in schoolchildren. We hypothesized that bright light therapy would have an additive effect on slowing myopia progression when combined with low-dose atropine. If proven effective, this combination treatment could represent a novel therapeutic strategy. Combination treatment, also known as polytherapy, has emerged as a new approach in recent years. The rationale for its use is that there may be an additive effect on treatment efficacy if the treatment of choice affects myopia pathogenesis through distinct mechanisms. A recent meta-analysis suggests that combination treatment is the most effective intervention for myopia control [30]. Both Tan et al [31] and Kinoshita et al [32] have shown that the combination of 0.01% atropine and orthokeratology is more effective than orthokeratology alone in slowing axial elongation. As the proposed myopia control mechanisms differ between atropine and bright light therapy, this randomized clinical trial is designed to investigate whether combining these 2 promising therapies results in an additive effect.

Additionally, bright light therapy has not yet been studied for its effectiveness in controlling myopia progression. Therefore, our results will contribute valuable insights to the literature regarding its potential efficacy. Another feature of this study is the administration of low-dose atropine twice daily. At the time of writing, all other randomized clinical trials [20,33-40] using low-dose atropine to control or prevent myopia only administered the treatment once daily. It has been repeatedly shown that 0.01% atropine is a safe and well-tolerated treatment but is limited in its effectiveness [34]. Increasing the concentration of atropine would lead to a stronger myopia control effect [34], but unfortunately, both the risk of adverse effects [34] and the extent of the rebound effect are dose dependent [38]. Administering atropine twice daily provides a more prolonged presence of the medication, which could result in a stronger myopia control effect without significantly increasing the risk of adverse events.

A recent 1-year–long randomized clinical trial by He et al [41] provides supporting evidence for this hypothesis. In their study, the axial elongation and spherical equivalent refraction progression in the twice-daily group (n=36) were 34% and 53% lower, respectively, than in the once-daily group (n=34). There were no clinically significant differences between the 2 groups in safety parameters, including accommodation amplitude, pupil diameter, and intraocular pressure. The study proposed in this paper, with a larger sample size and longer follow-up, will provide additional insights into optimizing the balance between efficacy and safety in low-dose atropine treatment for myopia control.

The strengths of this study include its prospective, randomized design and the use of masked examiners. The primary limitation of this study is the lack of masking of participants. The study duration is only 24 months due to budget constraints, so longer-term results will not be available. In addition, while the effectiveness of bright light therapy is examined in this study, further research is still needed to determine the optimal light level. Whether the benefit, if any, would apply to patients with different characteristics also remains to be assessed.

The results of this study will be disseminated at scientific conferences and in peer-reviewed, indexed journals in the fields of optometry and ophthalmology.

## Supplementary material

10.2196/90893 Checklist 1 Peer review report by the Research Impact Fund Committee, University Grants Committee, Research Grants Council (Hong Kong).

## Abbreviations

eCRF: electronic case report form
REDCap: Research Electronic Data Capture
SPIRIT: Standard Protocol Items: Recommendations for Interventional Trials
