# Supplementary material for: Effectiveness of Low-Dose Atropine Combined With Bright Light Therapy for Controlling Myopic Eye Growth in Schoolchildren: Study Protocol for a Randomized Controlled Trial
Source: JMIR Res Protoc. 2026 Apr 13;15:e90893. doi: 10.2196/90893 (PMC13075465; doi:10.2196/90893)
Supplement: Checklist 1 [file resprot-v15-e90893-s001.pdf]

Notes for the Applicants
Research Grants Council's Policy on
Providing Feedback to Applicants

It is the policy of the Research Grants Council (RGC) to provide all the applicants with the comments (except those that may reveal the reviewer's identities) on the research proposals considered in the Research Impact Fund (RIF). The purpose of sharing the comments is to help researchers improve and refine their research methodologies and provide Hong Kong with an inflow of valuable ideas and expertise from the international academic community.

The RGC's funding decisions are based on the careful assessments and considered judgment of the RIF Committee (RIFC). It should be noted that the final funding decision can be influenced by the following factors:

(a) limited amount of funding available for the RGC to distribute; and/or
(b) keen competition.

The recommendations made by individual reviewers and members of the RIFC are considered not strong and justifiable enough for funding to be awarded or not. It is inappropriate to assume that any subsets of comments from individual reviewers and members of the RIFC are specifically responsible for the final funding decision. Against this background, the funding decisions are final and no appeal will be considered unless in case of a major procedural flaw.

Research Grants Council
January 2019

RGC Research Impact Fund 2018/19 Exercise

Proposal No. R5032-18F

Proposal Title: Effectiveness of Bright Light Therapy, Myopic Defocus, Atropine and the Combinations for Controlling Myopic Eye Growth in Schoolchildren: A Randomized Control Trial

General comments on the academic merits of the proposal

Overall comments on academic merits of the proposal:

The trial is well thought out, has a sound evidential basis for each aspect of the intervention, includes most of the controls necessary to answer the key questions for clinicians, and is to be carried out by a team that has all of the skills necessary for it to be a success. All the approaches have a solid basis in animal studies or in correlational data, but the project will be novel in directly assessing these approaches together in a clinical human population.

In Summary a promising and very good proposal showing novel approaches to an issue of global interest, pushing the competitiveness of Hong Kong research.

Strengths:

The concept of the study is well-designed and solid. It serves a broad community both in health and financial aspects in the long run.

Weaknesses:

No clear information about recruitment of children and a more detailed plan of how to maintain children's compliance with respect to the complex and various treatments.

Suggested improvements:

The proposal would benefit from the addition of additional study groups, looking at different combinations of the clinical interventions.

I agree with one of the reviewers that additional alternative methods for the validation of patient compliance especially with respect to the complexity of the proposed approach due to the need for daily therapeutic interventions should be considered for the experimental design and methodology. The applicant should develop a plan to include a study group subjected to all three therapeutic interventions as polytherapy.

General comments on potential research impacts of the proposal

This is research directly addressing a major public health issue. If successful, the project has the potential for a major advance in one of the commonest problems of eye health, with a strong health-economics justification since it will have a lifelong effect following therapies in middle childhood. Benefits will be most evident in Hong Kong and other urbanized East Asian context, but potentially have worldwide application.

***

General comments on the academic merits of the proposal

Overall comments on academic merits of the proposal:

I have carefully read the proposal as well as the comments by the different reviewers. The idea of developing newer tools to limit Myopia in children is both timely and the approach taken by the team is appropriate. The questions that I had raised during the review of the preliminary proposal have also been addressed to my satisfaction. The results of this study will not only have local relevance, but also impact practice of prevention on other countries.

Strengths:

The team of investigators, their track record and the clarity of the plan are all strengths.

Weaknesses:

While, the investigators argue that cost prohibits from doing a combined study of the different interventions. This would have a tremendous

impact - if there is a way to get the project funded fully.

General comments on potential research impacts of the proposal

The research will impact practice of myopia prevention both locally at Hong Kong and internationally.

***

General comments on the academic merits of the proposal

Overall comments on academic merits of the proposal:

A well written and clear proposal that is feasible and likely to generate immediate benefit for young patients in the Asian region, and potentially more broadly.

Strengths:

A novel approach to evaluate effect of bright light therapy, alone and with complementary approaches. The applicants have strong a synergistic research skills and provide a strong scientific basis for the proposed therapy. The project exploits the unique opportunities in Hong Kong to attack this problem.

Weaknesses:

Omission of study group employing to all three therapeutic interventions. This a critical missing part of the experimental approach. Compliance of young research subjects may be an issue, control for degree of myopia on entering study. No control for the amount of time children spend out of doors in bright sunlight.

Suggested improvements:

1. Include study using all interventions.
2. Provide outdoor light control.

General comments on potential research impacts of the proposal

The results of this study, if positive, will be immediately applicable to myopia therapy, alone or in conjunction with existing drug and contact

lens therapies.

***

Additional Comments

Given the pervasive use of computers and handheld digital devices, eye health is at risk especially with the digital native generation. This is research directly addressing a major public health issue, and with strong health and economic benefit to society. What I am not so clear is the immediate near term impact, but overall, it has both broad reach and high mid and long term impact.

***

While the research topic - prevention and control of Myopia for children is of vital importance to public, and it has significant impact worldwide in the long term but it lacks of a concrete proposal on how to solve the problem. There is a flavour of trial and error and heuristic approach is adopted.

Proposal Ref.No. PolyU R5032-18F

Research Impact Fund
External Reviewer's Assessment Form

Proposal Title: Effectiveness of Bright Light Therapy, Myopic Defocus, Atropine and the Combinations for Controlling Myopic Eye Growth in Schoolchildren: A Randomized Control Trial

Project Coordinator: Prof To, Chi-ho
University of PC: PolyU

SECTION A : DETAILED COMMENTS

1. Please rate and comment on the academic merits and science content of the proposal.

Excellent	Very Good	Good	Fair	Poor
☑	☐	☐	☐	☐

Comments:
The proposed work is based on two hypotheses, namely that intervention using bright light therapy inhibits myopia progression in school-age children when employed during reading and homework at home. The second hypothesis entails that the approach of the first hypothesis when combined with other myopia-mitigating approaches, specifically a pharmacological and an optical intervention, results in a more effective combination therapy approach.

The academic merits and science content of the proposal are competitive with respect to its individual components and approaches. However, the highly innovative synergistic integration of such individual concepts into a well-developed prospective clinical trial supported by a wealth of previously published work and preliminary data as well as by impactful clinical studies from the applicant's field and other subspecialties of medicine targeting relevant mechanisms moves this proposal into the excellent range.

The stated objectives while not interdependent are designed in a straightforward placebo-controlled blinded manner to inform each other towards meaningful clinical and also adequately designed mechanistic analyses using state of the art technologies and procedures.

The applicant clearly identifies how novel properties of the therapeutic interventions and their combinations will be assessed, validated and analysed in a clinically relevant and experimentally rigorous manner. Resulting data sets, especially those of objectives 1 and 2 of the proposal, are likely to contribute significantly to the development of novel clinical practice guidelines.

Validation of data sets resulting from clinical studies using an adequately powered study design is adequately considered and effectively incorporated in the experimental design of all three objectives.

The approaches to discover novel properties of the proposed therapeutic interventions, and especially of the proposed combination treatments with respect to safety and efficacy, are adequately controlled to enable identifying potentially confounding factors not targeted by the experimental rationale of the study.

The description of previous work in the field, such as preclinical and clinical data, and its relevance to the

proposed project is adequate and supports the study's rationale.

A more detailed plan for the validation of patient compliance especially with respect to the complexity of the proposed approach due to the need for daily therapeutic interventions would further strengthen the experimental design and methodology.

The omission of a study group subjected to all three therapeutic interventions as polytherapy due to financial constraints reduces the overall merit of the study.

2. Please rate and comment on the long term goal of the proposal and its potential to develop into an area of strength.

Excellent	Very Good	Good	Fair	Poor
☑	☐	☐	☐	☐

Comments:
The central goal of this proposal seeks to develop novel more effective intervention strategies to inhibit myopia progression in school-age children when employed during reading and homework at home using bright light therapy combined with other myopia-mitigating approaches, specifically a pharmacological and an optical intervention. The proposal addresses interesting aspects of this area of research in a logical and rigorous manner. The objectives of the proposal as presented will likely adequately achieve the stated goals of the project. The proposed research agenda is likely to produce a combination of descriptive clinical data with respect to the stated objectives. In particular and supported by adequate preliminary data, the proposed research agenda will result in effective technology translation and innovative treatment options for affected patients developing the project into a significant area of strength.

3. Please rate and comment on the opportunities for effective synergism among the participating researchers, research groups, universities and partners.

Excellent	Very Good	Good	Fair	Poor
☑	☐	☐	☐	☐

Comments:
The multidisciplinary research team is comprised of optometrists, public health experts, a biologist, and an ophthalmologist, all uniquely situated to contribute valuable expertise to a highly synergistic team addressing the challenging epidemic of myopia in school-age children. Since myopia research is an area of emphasis at the Hong Kong Polytechnic University and its partners at the University of Hong Kong and Australian National University there is outstanding synergism among caregivers, health professionals, researchers, technology developers, and a range of other academic stakeholders and university partners. Altogether, this significantly supports the feasibility of the proposed work and the likelihood of success.

4. Please rate and comment on the viability of the proposal, particularly in terms of its project management. The project team's ability to put in place an effective governance structure to ensure prudent deployment of

resources would be important considerations.

Excellent	Very Good	Good	Fair	Poor
☑	☐	☐	☐	☐

Comments:
With the overall positive rationale and significance to do research in the field of therapy development for the challenging epidemic of myopia in school-age children the project provides a well-developed and highly feasible work plan for how the design and characterization of the proposed therapeutic interventions and their combinations, as well as related data acquisition, can be accomplished. Both focus of the experiments and experimental strategies especially with respect to adequate clinical controls enhance feasibility. The project team proposes to seek professional input from colleagues in the School of Public Health at the University of Hong Kong to optimize study design and logistics. Management expertise related to the team and its constituents, patient recruitment and retention, collaborations with academic partners resulting in high-impact data, clear prioritization strategies for both the mechanistic and clinical analyses, clearly identifies the project team's ability to put in place an effective governance structure to ensure prudent deployment of resources as outstanding. Previous and ongoing effective use of resources with the support from funding agencies identifies the project coordinator of this collaborative research proposal as highly capable of establishing a research program comprised of multiple university facilities and areas of complementary expertise. With previous support, the team was already able to significantly advance the program, particularly with respect to the successful optical intervention technology. All of these are additional promising indicators of the ability to generate an effective governance structure to ensure prudent deployment of resources.

5. What do you consider to be the most original or innovative aspect of the proposed research? What advances would the research result bring about to the related field if the proposed research is successful?

Comments:
Design and characterization of novel therapeutic interventions and their combinations for the challenging epidemic of myopia in school-age children have outstanding intrinsic merit. The study as designed will likely yield relevant and impactful data beyond already published results for part of the included mono-therapies and will generate a better mechanistic focus for the field through maximization of available output parameters in objectives one and two as well as through utilizing adequate validation methods. The straightforward combination of therapeutic interventions into a potentially very impactful, safe, and more effective poly-therapy and rigorously testing its clinical use has very high intrinsic merit. The study as designed will likely yield highly novel and impactful data beyond already published results and will generate a better clinical practice focus for the field through maximization of available output parameters in all objectives through utilizing adequate validation methods.

6. Please comment on the reasonableness of the proposed budget and manpower planning and project duration. [Mandatory matching funds are required from UGC-funded universities / partners for approved projects on a 70%(RGC) / 30%(university / partner(s)) basis.]

Comments:

The budget appears reasonable and also with respect to manpower planning and project duration. Mandatory matching fund rates required from UGC-funded universities / partners for approved projects on a 70% (RGC) / 30% (university) basis have been met adequately.

7. Overall Comments

Overall comments on academic merits of the proposal:
The academic merits and science content of the proposal are competitive with respect to its individual components and approaches. However, the highly innovative synergistic integration of such individual concepts into a well-developed clinical study supported by a wealth of previously published work and preliminary data as well as by preclinical studies targeting relevant mechanisms moves this proposal into the excellent range.

Strength:
The applicant clearly identifies how novel properties of the therapeutic interventions and their combinations will be assessed, validated and analysed in a clinically relevant manner. Resulting data sets, especially those of objectives 1 and 2 of the proposal, are likely to contribute significantly to mechanistic insights and novel clinical standards of care.

Weaknesses:
A more detailed plan for the validation of patient compliance especially with respect to the complexity of the proposed approach due to the need for daily therapeutic interventions would further strengthen the experimental design and methodology. The omission of a study group subjected to all three therapeutic interventions as polytherapy due to financial constraints reduces the overall merit of the study.

Suggested improvements:

Additional alternative methods for the validation of patient compliance especially with respect to the complexity of the proposed approach due to the need for daily therapeutic interventions should be considered for the experimental design and methodology. The applicant should develop a plan to include a study group subjected to all three therapeutic interventions as polytherapy, which might require working with funding agencies towards accomplishing this critical missing part of the experimental approach.

SECTION B : SUMMARY OF ASSESSMENT

(1) General Comments on Potential Research Impacts

The proposed research agenda is likely to produce a combination of descriptive and clinical data with respect to the stated objectives. In particular, and supported by adequate preliminary data, the proposed research agenda will identify and quantify the efficacy of novel intervention strategies inhibiting myopia progression in school-age children. The integrated approach combining medical and public health research approaches with intervention and mechanistic analyses has a good potential to produce novel impactful data sets potentially leading to more effective poly-therapy-based treatments for myopia with short-term beneficiaries being the regional population, and mid-term and long-term beneficiaries being patients suffering from developing myopia internationally and accompanying commercial implementation at the respective levels.

Proposal Ref.No. PolyU R5032-18F

Research Impact Fund
External Reviewer's Assessment Form

Proposal Title: Effectiveness of Bright Light Therapy, Myopic Defocus, Atropine and the Combinations for Controlling Myopic Eye Growth in Schoolchildren: A Randomized Control Trial

Project Coordinator: Prof To, Chi-ho
University of PC: PolyU

SECTION A : DETAILED COMMENTS

1. Please rate and comment on the academic merits and science content of the proposal.

Excellent	Very Good	Good	Fair	Poor
☑	☐	☐	☐	☐

Comments:
Myopia is an important public health issue in many Asian countries. This is a carefully planned study comparing some of the most successful treatment options for controlling myopia progression (alone and in combination).

2. Please rate and comment on the long term goal of the proposal and its potential to develop into an area of strength.

Excellent	Very Good	Good	Fair	Poor
☑	☐	☐	☐	☐

Comments:
The research team has a strong history of leading myopia research, not only in Hong Kong but internationally. The proposed project builds on their current strengths.

3. Please rate and comment on the opportunities for effective synergism among the participating researchers, research groups, universities and partners.

Excellent	Very Good	Good	Fair	Poor
☑	☐	☐	☐	☐

Comments:
The research team has a strong record of collaborations in both basic research into myopia and in the successful completion of randomized clinical trials of myopia treatments. The team has a good mix of skills.

4. Please rate and comment on the viability of the proposal, particularly in terms of its project management. The project team's ability to put in place an effective governance structure to ensure prudent deployment of

resources would be important considerations.

Excellent	Very Good	Good	Fair	Poor
☑	☐	☐	☐	☐

Comments:
The research team has successfully conducted similar trials in the past. They are familiar with the management of children undergoing myopia treatments with contact lens and atropine. The governance structure is appropriate.

5. What do you consider to be the most original or innovative aspect of the proposed research? What advances would the research result bring about to the related field if the proposed research is successful?

Comments:
Various strategies for controlling myopia progression are used in clinical practice (eg. soft multifocal contact lenses, atropine, orthokeratology, increased outdoor light exposure, multifocal spectacles). There is good evidence on the efficacy of these treatments when used alone, but virtually no data on the combined effectiveness of these treatments. Combination therapies do provide an exciting opportunity to improve the management of myopia and this study will answer some important questions on this topic. The results can be directly translated into clinical practice to aid in myopia management of children.

6. Please comment on the reasonableness of the proposed budget and manpower planning and project duration. [Mandatory matching funds are required from UGC-funded universities / partners for approved projects on a 70%(RGC) / 30%(university / partner(s)) basis.]

Comments:
The budget is very reasonable. A multinational company running a similar study with 608 children for 2 years would expect the study to cost >US$10M. The number of staff to be employed seems appropriate.

7. Overall Comments

Overall comments on academic merits of the proposal:

Strength: Addresses an important public health problem. Good study design. Appropriate research team and budget.

Weaknesses: Maintaining children's compliance with complex treatments will be challenging. Using light box therapy instead of outdoor light exposure is based on SAD symptoms and not myopia treatment efficacy.

Suggested improvements: A management strategy for how children will be managed at the end of the 2 yr trial – will they be offered the most effective treatment strategy.

Proposal Ref.No. PolyU R5032-18F

SECTION B : SUMMARY OF ASSESSMENT

(1) General Comments on Potential Research Impacts

The study outcomes can translate directly into clinical practice for the management of myopia in children. Will be important for clinical practice, policy and industry. Potential for commercial applications.

Proposal Ref.No. PolyU R5032-18F

Research Impact Fund
External Reviewer's Assessment Form

Proposal Title: Effectiveness of Bright Light Therapy, Myopic Defocus, Atropine and the Combinations for Controlling Myopic Eye Growth in Schoolchildren: A Randomized Control Trial

Project Coordinator: Prof To, Chi-ho
University of PC: PolyU

SECTION A : DETAILED COMMENTS

1. Please rate and comment on the academic merits and science content of the proposal.

Excellent	Very Good	Good	Fair	Poor
☐	☑	☐	☐	☐

Comments:
The current proposal builds on work by the investigators and others over many years on the possible methods to limit the progression of myopia. It takes techniques that have been proven to have some capacity to limit myopia, and newer techniques such as Bright Light Therapy, and looks at ways to possibly optimize those effects. Given that there is still no optimal answer on the most effective strategy to limit myopia progression, there is a good basis for this proposal.

2. Please rate and comment on the long term goal of the proposal and its potential to develop into an area of strength.

Excellent	Very Good	Good	Fair	Poor
☐	☑	☐	☐	☐

Comments:
The long term and short term goals of the proposal are clearly explained and are reasonably straight forward. The research design is logical and appropriate to the questions being addressed, and there is a reasonable expectation that this research topic will remain an area of strength given that most of the methodologies being employed are well established ones. The inclusion of Bright Light Therapy further enhances the value of the proposal.

3. Please rate and comment on the opportunities for effective synergism among the participating researchers, research groups, universities and partners.

Excellent	Very Good	Good	Fair	Poor
☑	☐	☐	☐	☐

Comments:
The backgrounds and expertise of the members of the research team provide a comprehensive range of

research skills appropriate to the research questions being addressed.

4. Please rate and comment on the viability of the proposal, particularly in terms of its project management. The project team's ability to put in place an effective governance structure to ensure prudent deployment of resources would be important considerations.

Excellent	Very Good	Good	Fair	Poor
☐	☑	☐	☐	☐

Comments:
Given that the techniques and protocols are based on previous studies by the investigators and others, there is every expectation that the work will be satisfactorily completed and lead to worthwhile information on limiting myopia progression. The logic behind the use of these interventions, singly and in combination, is well described. The project team has diverse interests and expertise, and are well-placed to conduct successful experimentation.

5. What do you consider to be the most original or innovative aspect of the proposed research? What advances would the research result bring about to the related field if the proposed research is successful?

Comments:
There has been considerable research conducted on the possible mechanisms to limit myopia progression, but this proposal to look not only at possible interventions but also combination treatments moves these investigations forward in a logical fashion. The inclusion of Bright Light Therapy, a newer intervention, further enhances the value of the research. The prospect of developing improved interventions to limit myopia progression is highly relevant. Given the high prevalence of myopia globally, and particularly in Asia, and the overall impact of myopia on communities as well as the potentially serious complications for individuals, the study outcomes will be of significance in the future management of myopia.

6. Please comment on the reasonableness of the proposed budget and manpower planning and project duration. [Mandatory matching funds are required from UGC-funded universities / partners for approved projects on a 70%(RGC) / 30%(university / partner(s)) basis.]

Comments:
The applicants already have in place much of the equipment and infrastructure required to complete this research. The proposed budget therefore seems reasonable and the staff requests are appropriate.

7. Overall Comments

Overall comments on academic merits of the proposal:

The development and progression of myopia is an issue of relevance and importance to both the individual and the community. The correction of myopia has significant costs to the community. High myopia can also lead to complications for the individual which can be debilitating. The occurrence of myopia has global implications, but it is particularly relevant within Asia.
The applicants have presented a research plan attempting to take research on interventions to limit the progression of myopia to a new level. The proposed research builds on both the research undertaken by the investigators and the significant amount of previous research by other investigators.

Strength:

The research design is clearly explained and is based on the published literature and the earlier research of the investigators and other researchers. The issue of myopia progression is a significant one with important implications for individuals and the community. The need for and value of a comprehensive study on interventions to limit the development and progression of myopia and strategies for its limitation are accepted. This study takes interventions previously established as having valuable roles, together with newer ones, and further examines ways of enhancing their outcomes.

Weaknesses:

A limitation of the current proposal is that an investigation of all the named interventions in combination could also be very helpful. However, the investigators have pointed out that the limitations of time and budget make that addition impractical at this stage.

Suggested improvements:

SECTION B : SUMMARY OF ASSESSMENT

(1) General Comments on Potential Research Impacts

Given the prevalence of myopia and the consequences for communities and individuals, successful implementation of strategies for limiting its progression will have strong research impacts.

Proposal Ref.No. PolyU R5032-18F

Research Impact Fund
External Reviewer's Assessment Form

Proposal Title: Effectiveness of Bright Light Therapy, Myopic Defocus, Atropine and the Combinations for Controlling Myopic Eye Growth in Schoolchildren: A Randomized Control Trial

Project Coordinator: Prof To, Chi-ho
University of PC: PolyU

SECTION A : DETAILED COMMENTS

1. Please rate and comment on the academic merits and science content of the proposal.

Excellent	Very Good	Good	Fair	Poor
☑	☐	☐	☐	☐

Comments:
Since recognising that topical atropine can slow or halt myopia progression, albeit with significant side effects, around 40 years ago, researchers have made incremental steps in developing a range of clinically acceptable interventions for myopia. At this stage, low dose atropine, multifocal 'defocusing' contact lenses and orthokeratology are the most promising interventions and these are all now being used clinically in an effort to slow the onset of the worldwide myopia epidemic, predicted by epidemiologists, which is already having an impact in areas of Asia. However, none of these interventions completely halts myopia development or progression and, despite the fact that researchers in the field have speculated over whether combinations of existing therapies might achieve an additive effect in myopia therapy, the field is, to date, lacking a high quality clinical trial to test the efficacy of combination interventions. The proposed study will fill this gap in our existing knowledge.

More recently, the recognition that regular, 'high light level' exposures, consistent with those experienced by children out-of-doors, are protective against myopia development have opened another potential avenue for intervention. There is now a need for study, such as this, to determine whether an active, light-based intervention can replace the experience of children simply spending more time outside, and to prove whether such an intervention is as effective as it has already been in animal models of myopia. Again, the proposed study will extend our knowledge in this area, which is of particular importance because, if successful, this approach would become the least invasive intervention for myopia progression available to clinicians.

In summary, the trial, as a whole, is well thought out, has a sound evidential basis for each aspect of the intervention, includes most of the controls necessary to answer the key questions for clinicians, and is to be carried out by a team that has all of the skills necessary for it to be a success.

2. Please rate and comment on the long term goal of the proposal and its potential to develop into an area of strength.

Excellent Very Good Good Fair Poor

☑ ☐ ☐ ☐ ☐

Comments:

My comments above, in 1., highlight the long term benefits to society of a successful outcome from this proposed project. In their proposal, the authors accurately highlight the public health risks of a myopia epidemic, as well as the benefits of developing effective interventions, and combinations of interventions, so I have nothing to add to their comments in this respect. Simply put, a successful outcome from the proposed project will certainly have a significant impact on the way that ophthalmic clinicians manage myopia into the future. The team behind this project already have a strong track record in this field, so to say whether this will develop into an area of strength is immaterial, as it is already an area of strength for them, and for their institution. It is also worth noting that the team contains members who have been central to the previous research work, in animals and humans, that has brought us to the stage at which a 'light therapy' intervention for myopia might be used in a clinical trial. This is of particular importance as as it is perhaps the only slight area of risk in the whole project as it is less easy to predict the outcomes.

3. Please rate and comment on the opportunities for effective synergism among the participating researchers, research groups, universities and partners.

Excellent	Very Good	Good	Fair	Poor
☑	☐	☐	☐	☐

Comments:

Large scale clinical trials of this type can only succeed through successful collaboration, and the PC has built a strong collaborative clinical trial group across multiple academic institutions in Hong Kong and Australia, and multiple research group within these institutions, professional bodies in Hong Kong, and both national and worldwide industry, including ophthalmic and Pharma R&D groups. The collaborators all bring important, and needed, skills and infrastructure to the clinical trial, which should ensure that the necessary synergism is present for its successful completion. The future opportunities for ongoing collaborations between individual and groups of researchers across these partners are substantial.

4. Please rate and comment on the viability of the proposal, particularly in terms of its project management. The project team's ability to put in place an effective governance structure to ensure prudent deployment of resources would be important considerations.

Excellent	Very Good	Good	Fair	Poor
☑	☐	☐	☐	☐

Comments:

The proposal is highly viable in the hands of the PC and the research collaborators brought in to the project. There can be no doubt that the team, collectively, has the discipline-specific expertise, breadth of knowledge and experience, 'manpower', and access to resources and infrastructure necessary to make a success of this project. I can see no gaps or areas of need in this respect. I have no doubts that the research team will work well together within the proposed project governance structure to ensure that the project is successfully completed.

5. What do you consider to be the most original or innovative aspect of the proposed research? What advances would the research result bring about to the related field if the proposed research is successful?

Comments:
As I state in 1., there are two original and innovative aspects to the proposed project: 1) The analysis of the potential of combination clinical interventions to control myopia development and progression; and 2) Proof of concept of a light therapy intervention for myopia. Success in either one or both of these areas will likely bring about highly significant public health benefits in respect of how the myopia epidemic is managed into the future. I would note that I agree with the researchers in that the proposal would benefit from the addition of additional study groups, looking at different combinations of the clinical interventions, but I understand that budget constraints in the grant scheme preclude the addition of these extra groups.

6. Please comment on the reasonableness of the proposed budget and manpower planning and project duration. [Mandatory matching funds are required from UGC-funded universities / partners for approved projects on a 70%(RGC) / 30%(university / partner(s)) basis.]

Comments:
The proposed budget and timeline looks entirely reasonable for a project of this type. As is to be expected, the overwhelming bulk of the budget is allocated to salaries for staffing the project and collecting and analysing the data, and to the cost of providing the interventions (i.e. contact lenses, spectacles, light boxes, drugs and placebos etc.). Some of this cost is offset by the University and research partners, consistent with the required 70/30 split.

7. Overall Comments

See my comments in the sections above., which I believe already adequately address my thoughts in terms of the sections below.

Overall comments on academic merits of the proposal:

Strength:

Weaknesses:

Suggested improvements:

RIF5

Proposal Ref.No. PolyU R5032-18F

SECTION B : SUMMARY OF ASSESSMENT

(1) General Comments on Potential Research Impacts

Again, I believe my responses above adequately cover my thoughts on the potential impacts of this research. Myopia is a worldwide problem, the impacts of which are particularly being felt in Asia at this stage, thus a successful outcome in this research project has significant impact in the long term on both a local and global scale.

Proposal Ref.No. PolyU R5032-18F

Research Impact Fund
External Reviewer's Assessment Form

Proposal Title: Effectiveness of Bright Light Therapy, Myopic Defocus, Atropine and the Combinations for Controlling Myopic Eye Growth in Schoolchildren: A Randomized Control Trial

Project Coordinator: Prof To, Chi-ho
University of PC: PolyU

SECTION A : DETAILED COMMENTS

1. Please rate and comment on the academic merits and science content of the proposal.

Excellent	Very Good	Good	Fair	Poor
☑	☐	☐	☐	☐

Comments:
The proposed project addresses a very important problem both specifically for Hong Kong eye health, and globally. The project is ambitious, testing not just one but three different combinations of bright light therapy with other approaches to alleviating the progression of myopia. All the approaches have a solid basis in animal studies or in correlational data, but the project will be novel in directly assessing these approaches together in a clinical human population. It is internationally important in exploiting the opportunities in Hong Kong for working with an East Asian population where this is an acute problem, in the context of a well developed public health and educational system.

2. Please rate and comment on the long term goal of the proposal and its potential to develop into an area of strength.

Excellent	Very Good	Good	Fair	Poor
☐	☑	☐	☐	☐

Comments:
If successful in reducing myopia, the project will be a very significant advance, both for clinical optometric practice and in enhancing scientific understanding of the pathways to myopia progression. Even if it cannot demonstrate a significant improvement, its design is sufficiently solid that it will have effectively advance the subject by indicating which therapeutic avenues continue to hold promise and which should be ruled out.

3. Please rate and comment on the opportunities for effective synergism among the participating researchers, research groups, universities and partners.

Excellent	Very Good	Good	Fair	Poor
☑	☐	☐	☐	☐

Comments:

The collaboration should be effective since most of the researchers are in the same institution with a record of past collaborations. Their expertise and professional capabilities are complementary in optometry, basic science, ophthalmology, and statistics. Dr Morgan is an external consultant whose expertise in this area of study is very high and whose guidance will be of great value. I am not in a position to judge however how actively he will collaborate in the planning, execution, and analysis of the programme.

4. Please rate and comment on the viability of the proposal, particularly in terms of its project management. The project team's ability to put in place an effective governance structure to ensure prudent deployment of resources would be important considerations.

Excellent	Very Good	Good	Fair	Poor
☑	☐	☐	☐	☐

Comments:
The proposal shows evidence of careful planning and team members already have experience in implementing large-scale clinical trials in the context of optometric practice with children.

5. What do you consider to be the most original or innovative aspect of the proposed research? What advances would the research result bring about to the related field if the proposed research is successful?

Comments:
The emphasis on bright light therapy is innovative. If successful it will provide a major new therapeutic approach to myopia progression, and make a substantial contribution to our scientific understanding of the mechanisms of this progression.

6. Please comment on the reasonableness of the proposed budget and manpower planning and project duration. [Mandatory matching funds are required from UGC-funded universities / partners for approved projects on a 70%(RGC) / 30%(university / partner(s)) basis.]

Comments:
The programme has been carefully planned and the budget well justified for a project of this scale.

7. Overall Comments

Overall comments on academic merits of the proposal:

Strength: 1. The novel approach to evaluation of bright light therapy, alone and in the context of complementary approaches. 2. The solid background of the applicants in the scientific basis of the proposed therapies. 3. The effective exploitation of the unique opportunities in Hong Kong to attack this problem.

Weaknesses: The proposal does not make clear just how the children will be recruited, any possible biases introduced by the recruitment procedure, and the range of levels of myopia which they expect in their sample.

Suggested improvements: A stratified design in terms of allocating age and level of myopic error to the different arms of the trial

RIF5

Proposal Ref.No. PolyU R5032-18F

SECTION B : SUMMARY OF ASSESSMENT

(1) General Comments on Potential Research Impacts

This is research directly addressing a major public health issue. If successful the project has the potential for a major advance in one of the commonest problems of eye health, with a strong health-economics justification since it will have a lifelong effect following therapies in middle childhood. Benefits will be most evident in Hong Kong and other urbanized East Asian context, but potentially have worldwide application.
